# Systematic investigation of a potential epidemiological and genetic association between male androgenetic alopecia and COVID‐19

**DOI:** 10.1002/ski2.72

**Published:** 2021-10-21

**Authors:** S. K. Henne, L. M. Hochfeld, C. Maj, M. M. Nöthen, S. Heilmann‐Heimbach

**Affiliations:** ^1^ Institute of Human Genetics School of Medicine & University Hospital Bonn University of Bonn Bonn Germany; ^2^ Institute for Genomic Statistics and Bioinformatics University of Bonn Bonn Germany

## Abstract

**Background:**

Male androgenetic alopecia (AGA) has been implicated as a putative risk factor in severe COVID‐19 based on high incidences of advanced AGA in male hospitalized COVID‐19 patients. Research further suggests that androgen signalling, which plays a central role in AGA aetiology, promotes SARS‐CoV‐2 infection and is associated with severe COVID‐19 symptoms in men.

**Objectives:**

We aimed to systematically investigate a potential association between AGA and COVID‐19 both on an epidemiological and a genetic level in a large single‐population cohort.

**Methods:**

We performed regression, genetic correlation and polygenic risk score (PRS) analyses using data from the UK Biobank and published GWAS data on AGA and COVID‐19.

**Results:**

Our analyses did not reveal any significant epidemiological or genome‐wide genetic association between AGA and severe COVID‐19. Pathway‐based PRS analyses however revealed a significant association in specific pathways, namely vitamin metabolism, natural killer cell‐mediated cytotoxicity, WNT signalling and aryl hydrocarbon receptor signalling.

**Limitations:**

We restricted our analyses to the white British population and used self‐reported AGA status. Sample size may be a limitation in our regression and PRS analyses.

**Conclusions:**

Our data yield no evidence for an epidemiological association between AGA and COVID‐19 but suggest that a shared genetic basis for both traits exists in specific pathways.

## 1 INTRODUCTION

Male androgenetic alopecia (AGA) is a highly heritable trait characterized by progressive hair loss in frontotemporal and vertex areas of the scalp AGA that has been implicated as a putative risk factor in severe COVID‐19 in a study by Wambier et al. that reported (i) worse COVID‐19 outcomes in hospitalized men with AGA in a non‐age‐corrected setting and (ii) higher incidences of advanced AGA in male hospitalized COVID‐19 patients compared to a reference population.^1^ Based on their findings, the authors suggest that baldness should be considered a risk factor called the ‘Gabrin sign’, named after the first doctor to die of COVID‐19 in the USA, Dr Gabrin. While these epidemiological data are based on limited numbers of individuals and used patient and control samples from different populations, research further suggests that androgen signalling, which plays a central role in AGA aetiology, facilitates SARS‐CoV‐2 infection via augmented ACE2 receptor expression and is associated with severe COVID‐19 symptoms in men,^2^ suggesting a potential biological link between the traits. This has prompted studies on the use of antiandrogens for the treatment of COVID‐19, which suggested beneficial effects such as reduced COVID‐19 severity and duration.^3^ Given these findings, we aimed to systematically assess a potential association between COVID‐19 severity and AGA on (i) an epidemiological and (ii) a genetic level using data from a single‐population sample from the UK Biobank. To this end, we performed regression analyses and genetic correlation and polygenic risk score (PRS) analyses using data from the UK Biobank and published GWAS data on AGA and COVID‐19 severity.

## 2 RESULTS AND DISCUSSION

Our cohort comprised a total of 6262 individuals (3101 men; 3161 women) who tested positive for SARS‐CoV‐2. Of these, 362 men and 194 women were hospitalized due to COVID‐19. Self‐reported AGA status was available on 3069 men. A total of 69% presented with AGA, with 23% classifying themselves as having frontotemporal balding, 26% frontotemporal and vertex balding and 19% complete baldness of the scalp. As no information on balding pattern was available for the women of this cohort, only men were included in the epidemiological association analyses. The median age (at time of the positive test result) of men was 68 (interquartile range 16), and the median age of women was 65 (interquartile range 15). The age distribution was expectedly higher in hospitalized individuals, with a median of 74 years (interquartile range 11) in both sexes.

**FIGURE 1:**
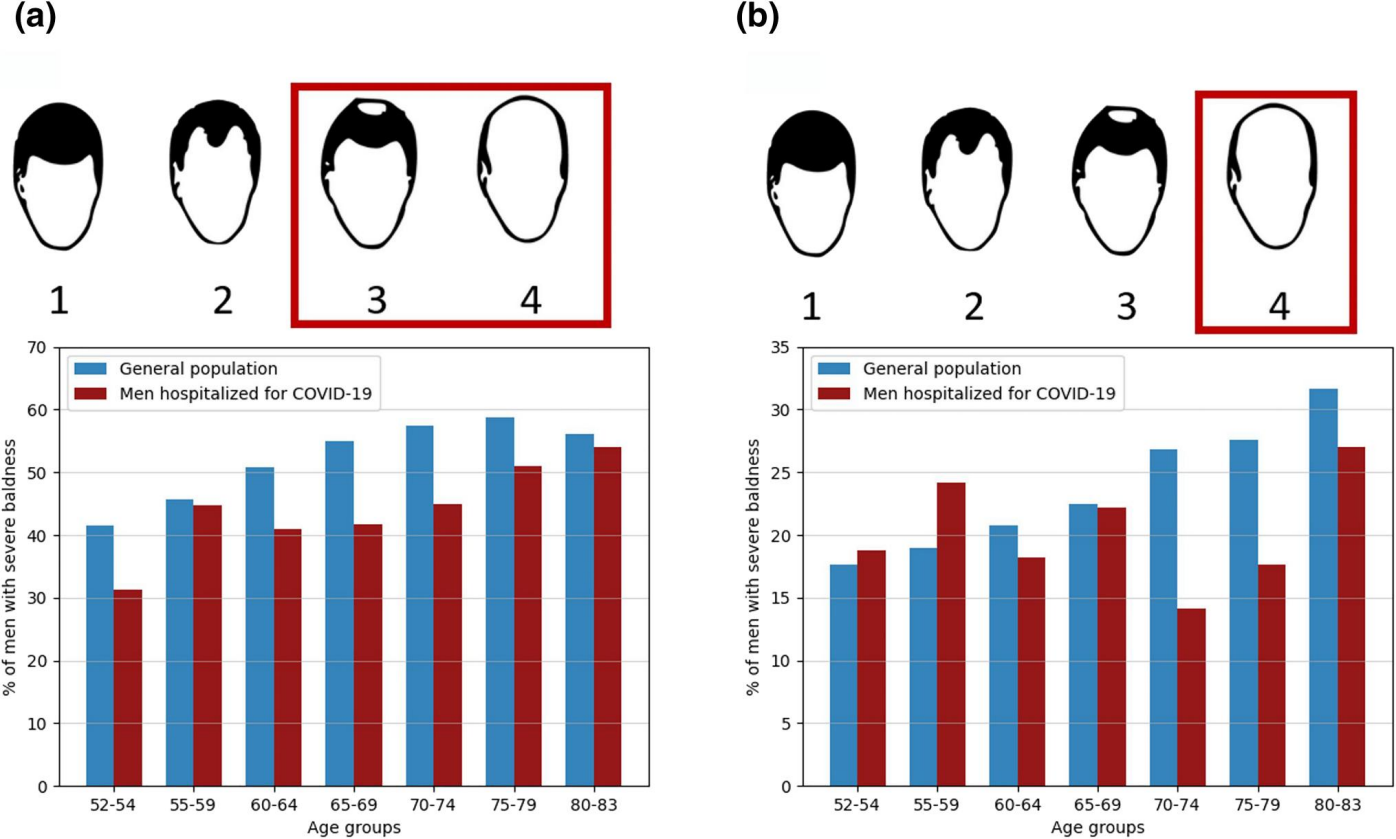
Prevalence of severe androgenetic alopecia (AGA) in men hospitalized for COVID‐19 compared to the general population across seven age groups from 52 to 83 in the UK Biobank. The data shown comprise 167 042 and 352 men in the UK Biobank general population and COVID‐19 hospitalization patients, respectively. Different definitions for severe AGA are applied per subplot. (a) Severe AGA is defined as UK Biobank patterns 3 and 4 and (b) severe AGA is defined as UK Biobank pattern 4

Our age‐corrected logistic regression analyses on AGA and COVID‐19 phenotypic data did not reveal any significant epidemiological associations between AGA and hospitalized versus non‐hospitalized COVID‐19 (*β* = −0.05, se = 0.05, *p* = 0.344). A slight trend towards a negative correlation between AGA and COVID‐19‐severity was seen when comparing the prevalence of severe AGA (defined as UK Biobank patterns 3 and 4) between hospitalized men and the UK male general population across six age groups between 52 and 83 (Figure 1a). Similarly, a recent cohort study of hospitalized COVID‐19 patients by Torabi et al. observed no association between COVID‐19 severity and AGA.^4^ These findings are, however, in contrast to previous reports by Wambier et al. that suggested a positive correlation between AGA and COVID‐19 severity. Notably, the prevalence of severe AGA (declared as ‘frontal and vertex’ balding) in the Australian general population used for comparison by Wambier et al.^1^ was much lower, ranging from 19% to 33% across age groups between 40 and 69. This discrepancy in AGA prevalence is likely due to the AGA scoring criteria used in the underlying study by Severi et al.^5^ While both Severi et al. and Wambier et al. declared the hair loss pattern to include Hamilton–Norwood scales IV–VII, the scoring of hair loss patterns by Severi et al. was performed using an accompanying pictogram depicting complete baldness of the scalp, identical to UK Biobank pattern 4. When defining severe AGA accordingly in the UK Biobank data, the prevalence of severe AGA was very similar between both the UK (18%–32%) and the Australian (19%–33%) general male populations (Figure 1b). Here, an increased ratio of severe AGA in hospitalized COVID‐19 patients is observed in the age groups 52–54 and 55–59, corroborating the findings of Wambier et al. Additional regression analysis for the age group 52–59, however, did not yield a statistically significant correlation between AGA and COVID‐19 severity (*β* = 0.22, se = 0.14, *p* = 0.12). It can therefore be assumed that the test statistics in the previously reported epidemiological association have been inflated by differences in AGA scoring criteria between case and control cohorts or a lack of age correction to account for the age dependency of both traits.

In line with our epidemiological data, no genetic correlation was observed between AGA and hospitalized COVID‐19 based on linkage disequilibrium score regression (LDSC) of genome‐wide association data (rg = 0.15, se = 0.10, *p* = 0.16). We also observed no association between hospitalized versus non‐hospitalized COVID‐19 and AGA‐PRS (*p* > 0.05). While LDSC and PRS analyses are powerful methods to detect a general shared genetic component between traits, these approaches have limited capacity to detect shared aetiological mechanisms in specific pathways and at specific genetic loci. These specific effects may be overlooked in a consideration based on the overall genetic contribution because of the limited share in this contribution. Thus, we aimed at further dissecting a potential genetic association between AGA and severe COVID‐19 by generating pathway‐based PRS (pPRS). Significant association of AGA‐pPRS with COVID‐19‐severity was found for four pathways: Vitamin metabolism (*p*_FDR_ = 0.02), natural killer cell (NKC)‐mediated cytotoxicity (*p*_FDR_ = 0.02) and WNT signalling (*p*_FDR_ = 0.02) in men as well as aryl hydrocarbon receptor (AhR) signalling (*p*_FDR_ = 0.02) in women. WNT signalling has been previously described in connection with both traits. It has long been recognized as a key factor in AGA aetiology^6^ and has been implicated in the formation of pulmonary fibrosis, a possible short‐ and long‐term complication of severe COVID‐19 infection.^7^ Vitamin metabolism may be interesting, as vitamin D deficiency has been linked to both AGA^8^ and acute respiratory distress syndrome severity.^9^ Neither AhR signalling nor NKC‐mediated cytotoxicity has been linked to AGA, though evidence exists linking them to COVID‐19. For instance, AhRs may be activated by SARS‐CoV‐2, contributing to COVID‐19 pathobiology and severity^9^ and NKC cytotoxic potential is significantly lowered in COVID‐19 patients admitted to the intensive care unit (ICU) compared to non‐ICU patients.^10^ Notably, aside from NKC cytotoxicity, all significantly correlated pathways have been previously described to interact with androgen signalling,^11^, ^12^, ^13^ emphasizing the aetiological relevance of androgen signalling for both traits. Why associations differ between males and females requires further investigation; however, hormone‐dependent effects of the associated pathways may play a role. These pathway‐based genetic associations between AGA and COVID‐19 severity may also lead to an epidemiological association between the traits. Given the fact that these overlapping pathways comprise only a very limited fraction of the pathways implicated in either AGA or COVID‐19, they, however, do not explain the strength of the previously reported epidemiological association. It is possible that a shared genetic component between both traits exists in additional pathways, which are not detected in this study due to the chosen *p*‐value cut‐offs of AGA‐associated single nucleotide polymorphisms (SNPs) in the PRS analyses. This study is further limited by the restriction of our epidemiological and PRS analyses to individuals of white British ancestry, thus requiring further testing in other ethnicities. As GWAS on AGA have been performed on individuals of white European ethnicity, this restriction was necessary to prevent bias in the pPRS analyses. This study is based on self‐reported AGA status with no evaluation or diagnosis by a physician, presenting another limitation. Generally, a larger sample size, particularly of hospitalized COVID‐19 patients, may improve the sensitivity and reliability of these analyses.

In summary, our data show no evidence for an epidemiological association between COVID‐19 severity and AGA. Detailed examination of the data that led to the previously reported conclusion that there is an epidemiological association between AGA and COVID‐19 suggests that the test statistics have been inflated by the use of different AGA scoring criteria between case and control cohorts or, in the case of the analysis on hospitalization outcomes, a lack of age correction. Correspondingly, we did not find any evidence for a general genetic correlation between the traits. However, our pPRS analyses suggest a shared genetic component between AGA and COVID‐19 in specific signalling pathways, posing interesting links between the pathophysiologies of both traits, where—even though not directly implicated by our pPRS analyses—androgen receptor signalling and crosstalk may act as the driving force.

## 3 MATERIALS AND METHODS

UK Biobank data on COVID‐19 test results, hospital inpatient admissions, AGA and imputed genotypes were used in these analyses (data last accessed on 16 December 2020). Severe COVID‐19 was assumed if the participant was hospitalized with COVID‐19 as a primary diagnosis. Non‐hospitalized participants tested positive for SARS‐CoV‐2 served as controls. AGA pattern was self‐reported based on pictograms on a scale of no balding (1), frontotemporal balding (2), frontotemporal and vertex balding (3) and complete baldness of the scalp (4). The most recent self‐report entry was used in this analysis.

### 3.1 Epidemiological correlation

We tested for an epidemiological correlation between severe COVID‐19 and AGA using age‐corrected logistic regression. To correct for the age dependency of AGA as well as COVID‐19‐related ospitalization, AGA patterns were first regressed on age at assessment using linear regression. Severe COVID‐19 was then regressed on the resulting AGA residuals, while correcting for age at hospitalization or most recent SARS‐CoV‐2 test. As the ratio of severe AGA (complete baldness of the scalp, UK Biobank pattern 4) prevalence between participants hospitalized for COVID‐19 and the general population was highest in the age groups of 55–59 and 52–54, we performed additional regressions for the age subset of 52–59.

### 3.2 Genetic correlation

We initially tested for a genome‐wide genetic overlap between AGA susceptibility and severe COVID‐19. Genome‐wide genetic correlation between AGA and COVID‐19 was assessed via LDSC using the LDSC tool.^14^ The analysis was performed on published GWAS summary statistics for AGA^15^ and for hospitalized versus non‐hospitalized COVID‐19 (COVID‐19HG, European cohorts excluding UK Biobank, version 5^16^).

### 3.3 PRS analyses

We established PRS based on SNPs associated with AGA (*p* < 5 × 10^−8^ and *p* < 5 × 10^−5^) in the AGA GWAS.^15^ These AGA‐PRS were calculated using PRSice‐2^17^ in sum‐calculation mode using autosomal and X‐chromosomal variants without regression on phenotype. Association of AGA‐PRS with severe COVID‐19 was subsequently tested in a sample of UK Biobank participants of white British ancestry. White British ancestry was assumed in individuals self‐identifying as White British, who show similar genetic ancestry in a principal component analysis of genotypes, as determined by the UK Biobank (data field 22 006). The final data set comprised 3101 men, of whom 362 were defined as cases (case–control ratio 1:7.6), and 3161 women, of whom 194 were defined as cases (case–control ratio 1:15.3). Logistic regression of severe COVID‐19 on AGA‐PRS was calculated separately per sex and *p*‐value cut‐off and corrected for age. We calculated pPRS by assigning SNPs to genes via positional mapping (distance <10 kb) and mapping genes to pathways using KEGG 2019, WikiPathways 2019 and Panther 2016 Enrichr libraries.^18^, ^19^ PRS were calculated as previously described. Logistic regressions of severe COVID‐19 on pPRS were performed per sex, *p*‐value threshold and library and corrected for age. Per iteration, significance of pPRS was corrected for multiple testing using the false discovery rate based on the included pPRS.

## CONFLICT OF INTEREST

The authors declare no conflict of interests.

## AUTHOR CONTRIBUTIONS

**S. K. Henne:** Data curation; Formal analysis; Writing – original draft; Writing – review & editing. **L. M. Hochfeld:** Conceptualization. **C. Maj:** Data curation; Formal analysis; Writing – review & editing. **M. M. Nöthen:** Project administration; Supervision; Writing – review & editing. **S. Heilmann‐Heimbach:** Conceptualization; Project administration; Supervision; Writing – original draft; Writing – review & editing.

## Data Availability

The findings of this study are supported by multiple datasets that are either available in public repositories or subject to third party restrictions. GWAS data for AGA and COVID‐19 are publicly available from the GWAS Catalogue (www.ebi.ac.uk/gwas/; study accession number GCST007020) and the Covid‐19 Host Genetics Initiative (www.covid19hg.org, release version 5, identifier B1_ALL_leave_UKBB), respectively. Third party restrictions apply to the availability of the UK Biobank dataset, which was used under licence for this study.
